# Knowledge and Awareness of Orthodontic Malocclusions and Optimal Treatment Timing Among Primary School Teachers in Andhra Pradesh

**DOI:** 10.7759/cureus.96202

**Published:** 2025-11-06

**Authors:** Aruna Dokku, Varikuti Angelina Praisy, Revathi Peddu, Devikanth Lanka, Saravanan Pichai, Ashok Kumar Talapaneni, Abirami Vetriselvan, Sanjeev Jakati

**Affiliations:** 1 Department of Orthodontics, Sibar institute of Dental Sciences, Guntur, IND; 2 Department of Orthodontics, Sibar Institute of Dental Sciences, Guntur, IND

**Keywords:** andhra pradesh, dental awareness, early intervention, oral health education, orthodontic malocclusion, orthodontics, school teachers

## Abstract

Background

Orthodontic malocclusions are common in school-aged children and, if left untreated, can lead to functional, esthetic, and psychosocial complications. Schoolteachers, as early observers of children's development, are well-positioned to recognize such issues. This study aims to assess the knowledge and awareness of primary school teachers in Andhra Pradesh regarding common orthodontic malocclusions and the importance of early intervention.

Methods

A cross-sectional questionnaire-based survey was conducted among 162 primary school teachers across various districts of Andhra Pradesh. The survey assessed participants’ ability to identify types of malocclusion, their understanding of the consequences of untreated malocclusion, and their knowledge about the appropriate timing for orthodontic treatment. Statistical analysis was performed to evaluate associations between awareness levels and demographic variables.

Results

The majority of teachers were able to recognize visible dental issues such as spacing (67%) and irregularly aligned teeth (63.5%). However, awareness was significantly lower for less obvious malocclusion types like crossbite, overbite, and underbite. Educational qualification showed a statistically significant association with awareness of crowding (p = 0.014) and spacing (p = 0.049). Younger teachers and those with fewer years of experience were more likely to know the recommended age for orthodontic consultation. Out of 162 participants, 60.5% correctly identified the appropriate age for the first orthodontic check-up, while 53.7% correctly responded about the best age to treat jaw-related malocclusions. However, only 44.4% were aware of the mixed dentition phase and its relevance. The overall awareness of complications resulting from untreated malocclusion was found to be low. Among these, only the association between malocclusion and tooth decay was statistically significant (p = 0.020).

Conclusion

The study highlights a moderate level of awareness among primary school teachers regarding orthodontic malocclusions, with notable gaps in knowledge about specific types and treatment timing.

## Introduction

Orthodontic malocclusions, encompassing both dental and skeletal anomalies, are prevalent among children and adolescents, significantly affecting oral health, facial aesthetics, and psychosocial well-being. Misaligned teeth and jaw discrepancies can lead to functional impairments such as speech difficulties and mastication problems, along with diminished self-esteem and social anxiety. Early recognition and timely referral of orthodontic problems during the mixed dentition period are essential to intercept developing malocclusions and reduce the need for complex treatment later. The mixed dentition stage offers a critical window for modifying growth patterns and correcting functional disturbances, thereby improving long-term occlusal stability and facial aesthetics. Studies have shown that early orthodontic correction during this period can effectively manage developing crossbites and other functional discrepancies, preventing skeletal asymmetries in adulthood [1]. Leveraging natural growth spurts during this period enables more effective, less invasive treatment outcomes and may reduce the future need for complex procedures like orthognathic surgery [2-4].

While parents and dental professionals are traditionally seen as key stakeholders in a child's orthodontic care, primary school teachers are uniquely positioned to observe early signs of malocclusion in day-to-day classroom interactions. Behaviours such as abnormal bite patterns, difficulty chewing, and speech impediments may be noticeable during routine activities. Despite this potential, limited research has examined teachers’ awareness of orthodontic issues. Existing studies suggest moderate knowledge of preventive orthodontics among teachers but reveal significant gaps in understanding optimal treatment timing and recognizing skeletal malocclusions [5-6].

Although parental awareness has been extensively studied, the role of teachers in early identification remains underexplored [7-9]. Increasing their awareness of common orthodontic problems, such as crowding, spacing, overbites, underbites, and crossbites, may enhance early diagnosis and timely referrals. The present study aims to quantitatively assess primary school teachers’ awareness regarding common orthodontic malocclusions and the appropriate timing for orthodontic referral, specifically measuring knowledge of the first orthodontic check-up and recognition of the mixed dentition stage as critical for early intervention.

## Materials and methods

The present study followed a cross-sectional survey design to assess the knowledge and awareness of primary school teachers regarding common orthodontic malocclusions and the importance of early intervention. The study was conducted among primary school teachers in the state of Andhra Pradesh, India. To ensure a representative sample, a multistage random sampling method was used. In the first stage, six districts (Visakhapatnam, Chittoor, Krishna, Guntur, Anantapur, and Srikakulam) were randomly selected out of the 26 districts in the state using a lottery method. In the second stage, three mandals from each of these districts were randomly chosen using a computer-generated list, representing a diverse mix of geographic and administrative areas. From each selected mandal, three primary schools were randomly picked in the third stage. Finally, in the fourth stage, three primary school teachers from each school were randomly selected using a random number table, resulting in a total sample of 162 teachers.

The sample size was calculated using the formula $n = \frac{Z^2 \, p \, (1 - p)}{d^2}$, where the assumed prevalence (p) of adequate awareness was set at 0.5, with a 95% confidence level (Z = 1.96) and a 5% margin of error (d = 0.05*). Teachers included in the study were those currently teaching classes from kindergarten to Grade 6 and who gave their informed consent to participate. Those who were on leave, not actively teaching during the data collection period, or who submitted incomplete questionnaires were excluded. The research protocol received approval from the Institutional Ethics Committee, Sibar Institute of Dental Sciences, Guntur (Approval No.: 445/IEC/SIBAR/2024), and participation was voluntary and anonymous, with data stored securely and accessed only by the research team.

A structured questionnaire (see Appendices) was designed in consultation with experts from orthodontics and public health to evaluate teachers’ knowledge of orthodontic problems. The questionnaire included 20 questions divided into three sections on demographic information, awareness of common malocclusions such as crowding, spacing, overbite, underbite, crossbite, and open bite, and understanding of the appropriate timing for orthodontic intervention. It also assessed factors that might influence knowledge, such as previous exposure to dental health workshops or information, and interest in professional development. The questionnaire was prepared in English, Telugu, and Hindi to accommodate language preferences and was pilot tested with 10 teachers for clarity and relevance. Data collection was carried out over five weeks (September - November 2024), with surveys provided in both online and paper formats depending on the accessibility and preference of the participants. Reminders were sent to ensure a high response rate and completeness of data. Of the 170 questionnaires distributed, the first 162 responses received in time were analyzed.

Statistical analysis

Statistical analysis of the data was done using IBM SPSS Statistics for Windows, version 26 (IBM Corp., Armonk, NY, USA) software package. Descriptive statistics, including frequency, percentage, mean, and standard deviation, were calculated for the various parameters. Significance tests were done using the chi-square test, the Wilcoxon Rank Sum test, and Mann Mann-Whitney U test. Significance was set at p <0.05.

## Results

A total of 162 primary school teachers (n=162) participated in the study. The sample consisted of female participants (n = 90, 55.6%) compared to males (n = 71, 43.8%). Most teachers were between the ages of 30-39 years (45.1%), with a mean age group of 31.35 years. All the demographic data is tabulated in Table 1.

**Table 1:** Demographic profile of participants (n = 162)

Variable	Categories	Frequency (n)	Percentage (%)
Gender	Male	71	43.8%
	Female	90	55.6%
	Not specified	1	0.6%
Age	20–29	70	43.2%
	30–39	73	45.1%
	40–49	19	11.7%
Years of teaching	0–5	102	63.0%
	6–10	47	29.0%
	11–15	6	3.7%
	15–20	7	4.3%
Educational qualification	Associate degree	97	59.9%
	Bachelor’s degree	24	14.8%
	Master’s degree	27	16.7%
	Doctorate	14	8.6%
Grade level taught	Kindergarten	17	10.5%
	Grades 1–2	34	21.0%
	Grades 3–4	21	13.0%
	Grades 5–6	90	55.5%

Analysis revealed a significant association between educational qualification and awareness of specific types of malocclusion. Awareness of crowding differed significantly across qualifications (p = 0.014), with the highest recognition among those holding Associate and Doctorate degrees. Similarly, awareness of spacing showed a statistically significant variation (p = 0.049), with the highest awareness among those with Master’s degrees (77.8%). No significant associations were found between educational level and awareness of overbite (p = 0.652), open bite (p = 0.557), crossbite (p = 0.230), or underbite (p = 0.083) (Table 2).

**Table 2:** Association between educational qualification and awareness of malocclusion types (n = 164) * Signifies statistically significant values (p < 0.05)

Type of malocclusion	Response	Associate (n=97)	Bachelor’s (n=24)	Master’s (n=27)	Doctorate (n=14)	Total (n=162)	p-value
Crowding	Yes	67 (69.1%)	15 (62.5%)	11 (40.7%)	10 (71.4%)	103	0.014*
	No	30 (30.9%)	9 (37.5%)	16 (59.3%)	4 (28.6%)	59	
Spacing	Yes	61 (62.9%)	17 (70.8%)	21 (77.8%)	9 (64.3%)	108	0.049*
	No	36 (37.1%)	7 (29.2%)	6 (22.2%)	5 (35.7%)	54	
Overbite	Yes	12 (12.4%)	3 (12.5%)	2 (7.4%)	1 (7.1%)	18	0.652
	No	85 (87.6%)	21 (87.5%)	25 (92.6%)	13 (92.9%)	144	
Open bite	Yes	1 (1.0%)	0 (0.0%)	0 (0.0%)	0 (0.0%)	1	0.557
	No	96 (99.0%)	24 (100.0%)	27 (100.0%)	14 (100.0%)	161	
Crossbite	Yes	4 (4.1%)	0 (0.0%)	0 (0.0%)	0 (0.0%)	4	0.230
	No	93 (95.9%)	24 (100.0%)	27 (100.0%)	14 (100.0%)	158	
Underbite	Yes	8 (8.2%)	1 (4.2%)	0 (0.0%)	0 (0.0%)	9	0.083
	No	89 (91.8%)	23 (95.8%)	27 (100.0%)	14 (100.0%)	153	

When evaluating knowledge of the ideal timing for orthodontic evaluation, 60.5% of participants correctly identified the appropriate age for the first orthodontic check-up, while 53.7% correctly responded about the best age to treat jaw-related malocclusions. However, only 44.4% were aware of the mixed dentition phase and its relevance (Table 3).

**Table 3:** Knowledge of ideal timing for orthodontic evaluation (n = 162)

Question asked	Correct response n (%)	Incorrect n (%)
Ideal age for first orthodontic check-up	98 (60.5%)	64 (39.5%)
Best age range to treat jaw-related malocclusions	87 (53.7%)	75 (46.3%)
Knowledge of mixed dentition phase and its significance	72 (44.4%)	90 (55.6%)

Factors potentially influencing knowledge and awareness were also assessed. Only 39.5% of teachers reported having attended a dental awareness workshop, while a much higher proportion (74.1%) expressed interest in learning more about orthodontics. However, just 35.8% felt confident in identifying malocclusions in children, and 43.2% had ever referred a child for dental evaluation, reflecting limited real-world application despite general interest (Table 4).

**Table 4:** Factors influencing knowledge and awareness (n=162)

Factor	Yes (n, %)	No (n, %)
Attended a dental awareness workshop	64 (39.5%)	98 (60.5%)
Interested in learning about orthodontics	120 (74.1%)	42 (25.9%)
Confident in identifying malocclusions	58 (35.8%)	104 (64.2%)
Have referred a child for dental issues	70 (43.2%)	92 (56.8%)

Demographic comparisons using chi-square analysis indicated no significant relationship between gender and orthodontic awareness (p = 0.281). Similarly, no significant association was observed between years of teaching experience and awareness (p = 0.166). Awareness levels appeared fairly uniform across all experience groups, suggesting that neither gender nor experience strongly influenced teachers’ orthodontic knowledge (Table 5).

**Table 5:** Comparison of awareness based on demographics (chi-square test)

Variable	Aware (n, %)	Not aware (n, %)	p-value
Gender			0.281
Male (n = 71)	43 (60.6%)	28 (39.4%)	
Female (n = 90)	55 (61.1%)	35 (38.9%)	
Years of experience			0.166
0–5 yrs (n = 102)	62 (60.8%)	40 (39.2%)	
6–10 yrs (n = 47)	27 (57.4%)	20 (42.6%)	
11–15 yrs (n = 6)	3 (50%)	3 (50%)	
15–20 yrs (n = 7)	4 (57.1%)	3 (42.9%)	

Lastly, the overall awareness of complications resulting from untreated malocclusion was found to be low. Among these, only the association between malocclusion and tooth decay was statistically significant (p = 0.020), underscoring the limited understanding among teachers of the broader health consequences of orthodontic issues (Table 6).

**Table 6:** Overall awareness of complications due to malocclusion among participants (n = 162)

Complication	Number aware (yes)	Percentage aware (%)	p-value
Jaw pain	37	22.8%	0.155
Biting problem	28	17.3%	0.245
Tooth decay	36	22.2%	0.020

## Discussion

The present study evaluated primary school teachers’ awareness and knowledge regarding orthodontic malocclusions, appropriate referral timing, and developmental dental phases. Our findings reveal notable gaps in knowledge, particularly regarding the ideal age for orthodontic evaluation and the significance of the mixed dentition phase, despite teachers being in a unique position to observe early signs of dental issues in children.

More than half of the teachers correctly identified the ideal age for a child’s first orthodontic check-up (60.5%) and the optimal time frame for treating jaw-related discrepancies (53.7%). However, knowledge regarding the mixed dentition phase, which is a critical window for early orthodontic intervention, was lacking in 55.6% of participants. Given that early identification and referral can prevent progression of malocclusion and reduce treatment complexity and cost, this lack of awareness is concerning.

Educational qualification was significantly associated with awareness of crowding and spacing malocclusions. Teachers exhibited greater awareness, particularly for spacing (p = 0.049), while knowledge of more complex anomalies like crossbite, underbite, and open bite remained low across all qualification levels. This aligns with findings by Rafighi et al. (2012), who observed that general knowledge of malocclusion types among non-dental professionals is typically limited to more visible anomalies such as crowding, whereas subtler conditions like open bite or crossbite are less recognized [10]. This indicates a potential gap in comprehensive dental education that prioritizes visible or commonly discussed issues over functional anomalies that may have long-term health implications. For instance, open bites and crossbites can lead to problems like jaw pain, speech difficulties, and uneven tooth wear, yet their importance might be underemphasized if not framed in terms of patient aesthetics.

Limited awareness of these specific types of malocclusion among non-dental professionals has been noted in multiple studies [10-12], emphasizing the need for foundational knowledge for educators who engage with young children regularly. This limited awareness may delay detection and referral for children who could benefit from timely orthodontic evaluation, especially for conditions like skeletal Class II and Class III malocclusions that require early intervention. Jain et al. (2021) emphasized the importance of school-based health programs as a major source of knowledge, highlighting the importance of “training the trainers” [13].

Interestingly, despite 74.1% of teachers expressing interest in learning more about orthodontics, only 35.8% felt confident in identifying malocclusions, and just 43.2% had ever referred a child for dental evaluation. This gap between interest and action highlights the need for structured training and awareness programs within school settings.

Although no significant associations were found between gender or years of teaching experience and orthodontic awareness. One noteworthy point to be considered in our study is that teachers with 0-5 years of experience showed higher knowledge levels, supported by the finding that 39.5% had attended workshops or programs on children's oral health. This contrasts with Shodan et al. (2014), who found experienced teachers to have greater knowledge about dental decay and malocclusion, as they might have gained knowledge through years of teaching experience [14]. The findings of this study show that less experienced teachers have more exposure to or better recall of modern orthodontic guidelines, while more experienced teachers may lack updated knowledge. This highlights the need for ongoing professional development and awareness programs to ensure educators, regardless of their years of service, are well-informed about the critical periods for orthodontic interventions. The enthusiasm of young teachers presents an opportunity to introduce regular professional development programs focusing on orthodontic issues.

When assessing awareness of complications associated with untreated malocclusion, very few teachers recognized potential consequences such as jaw pain (22.8%), biting problems (17.3%), or tooth decay (22.2%). Notably, only the awareness of tooth decay reached statistical significance (p = 0.020). Given the well-established associations between malocclusion, plaque accumulation, and subsequent caries risk, this lack of knowledge may hinder timely referrals and reinforce the need for cross-disciplinary collaboration between dental professionals and educators.

Overall, the study underscores the necessity of integrating oral health awareness into teacher training and continuing education. Teachers serve as front-line observers of children’s health and can play a pivotal role in early detection and referral if appropriately trained. Future initiatives should include periodic dental screening camps in schools, orientation sessions on dental milestones, and simple educational materials designed for non-dental professionals.

The Orthodontic Treatment Index (OTI) or Index of Orthodontic Treatment Need (IOTN) provides a structured approach to identifying malocclusion severity and the necessity for treatment. Familiarizing teachers with simplified principles from this index could help them recognize more severe cases and prioritize referrals. Such frameworks underscore the importance of timely orthodontic treatment, particularly for cases that pose functional or aesthetic concerns.

Effective dental education, particularly orthodontic malocclusions for children, can be achieved through a multidisciplinary approach, where teachers act as a strong pillar of oral health educators. However, their effectiveness depends on possessing adequate knowledge, a positive attitude, and regular brushing up of their dental knowledge by attending workshops and lectures about awareness.

Limitations

This study, though multicentric and methodologically robust, has certain limitations. Being cross-sectional, it reflects associations rather than causation. The findings are specific to Andhra Pradesh and may not be generalizable to other regions. Self-reported responses may have introduced some reporting bias. Future studies involving larger and more diverse populations with validated tools could provide broader insights.

## Conclusions

The present study revealed moderate awareness among primary school teachers in Andhra Pradesh regarding common orthodontic malocclusions and the importance of early referral, with notable gaps in understanding of the mixed dentition phase and treatment timing. Teachers, being in daily contact with children, can play a pivotal role in identifying early signs of malocclusion when equipped with appropriate knowledge.

These findings highlight the need to integrate basic oral health and orthodontic awareness into teacher training and continuing education programs. Strengthening collaboration between dental professionals and educators through school-based initiatives and periodic workshops can promote timely referrals and improve children’s oral health outcomes. Future studies with larger and more diverse populations, supported by validated assessment tools, are recommended to further substantiate and expand upon these findings.

## Appendices

Questionnaire

Section 1: Demographics

1. Gender:

☐ Male

☐ Female

☐ Prefer not to say

2. Age:

☐ 20-29

☐ 30-39

☐ 40-49

☐ 50-59

☐ 60+

3. Years of teaching experience:

☐ 0-5 years

☐ 6-10 years

☐ 11-15 years

☐ 16-20 years

☐ 21+ years

4. Grade level taught:

☐ Kindergarten

☐ 1st-2nd grade

☐ 3rd-4th grade

☐ 5th-6th grade

5. Educational background:

☐ Associate’s degree

☐ Bachelor’s degree

☐ Master’s degree

☐ Doctorate

Section 2: Awareness of Common Orthodontic Malocclusions

6. Which of the following terms are you familiar with as common orthodontic issues? (Check all that apply)

☐ Crowding of teeth

☐ Spacing between teeth

☐ Overbite

☐ Underbite

☐ Crossbite

☐ Open bite

☐ Jaws-related problems

☐None of the above

☐ Other (please specify): ___________

7. In which of the following situations would you recommend that a child be evaluated by an orthodontist? (Check all that apply)

☐ A child with overlapping or crooked teeth (crowding)

☐ A child with noticeable gaps between teeth (spacing)

☐ A child whose upper teeth significantly overlap the lower teeth (overbite)

☐ A child whose lower teeth are in front of the upper teeth when biting down (underbite)

☐ A child whose teeth do not align properly when the jaws are closed (crossbite)

☐ A child whose front teeth do not touch when the back teeth are together (open bite)

☐ A child whose jaws are improperly placed (skeletal)

☐ A child whose facial profile is not normal (esthetics)

☐ Not sure

8. What is the potential long-term impact if a malocclusion if not corrected?

☐ Jaw pain or TMJ (temporomandibular joint) disorders

☐ Difficulty in biting or chewing

☐ Increased risk of tooth decay

☐ No impact on dental health

☐ All of the above

9. Do you feel that orthodontic issues will impact the child's social behavior?

☐ Yes

☐ No

☐ Not sure

If yes, have you encountered such students in your class anytime? Explain.

10. Do you feel that orthodontic issues will impact the child's academic performance?

☐ Yes

☐ No

☐ Not sure

If yes, have you encountered such students in your class anytime? Explain.

11. How did you learn about these orthodontic issues? (Check all that apply)

☐ Personal experience

☐ Family or friends

☐ Professional development courses

☐ School health programs

☐ Media (TV, internet, magazines)

☐ Other (please specify): ___________

Section 3: Knowledge of Timing for Orthodontic Treatment

12. At what age do you think children should have their first orthodontic evaluation?

☐ 3-5 years

☐ 6-7 years

☐ 8-9 years

☐ 10-12 years

☐ 13+ years

☐ Not sure

13. When do you think is the ideal time for orthodontic intervention for the correction of jaw-related problems?

☐ Early childhood (3-6 years)

☐ Mixed dentition phase (6-12 years)

☐ Adolescence (12+ years)

☐ Not sure

14. Do you believe early orthodontic intervention can prevent more severe issues later?

☐ Yes

☐ No

☐ Not sure

Section 4: Factors Influencing Awareness and Knowledge

15. Have you attended any workshops or training sessions on children’s dental health?

☐ Yes

☐ No

16. How often do you receive information about children’s dental health at your school?

☐ Regularly (at least once a year)

☐ Occasionally (every few years)

☐ Rarely or never

17. Would you be interested in professional development opportunities focused on children’s dental health, including orthodontic issues?

☐ Yes

☐ No

18. Do you feel confident in recognizing orthodontic issues in your students?

☐ Very confident

☐ Somewhat confident

☐ Not confident

19. How often do you discuss dental health, including orthodontic issues, with your students or their parents?

☐ Often

☐ Sometimes

☐ Rarely or never

Section 5: Additional Comments

20. Please provide any additional comments or suggestions about how awareness of orthodontic issues can be improved among primary teachers.
